# Association of *HOTAIR* polymorphisms *rs4759314* and *rs920778* with cancer susceptibility on the basis of ethnicity and cancer type

**DOI:** 10.18632/oncotarget.9608

**Published:** 2016-05-26

**Authors:** Qichao Qi, Jiwei Wang, Bin Huang, Anjing Chen, Gang Li, Xingang Li, Jian Wang

**Affiliations:** ^1^ Department of Neurosurgery, Qilu Hospital of Shandong University and Brain Science Research Institute, Shandong University, Jinan, 250012, China; ^2^ Department of Biomedicine, University of Bergen, Bergen, 5009, Norway

**Keywords:** cancer, genetic susceptibility, HOTAIR, polymorphism

## Abstract

Polymorphisms in the *HOX* transcript antisense intergenic RNA (*HOTAIR*) have been recently associated with susceptibility to different cancers. Here, a meta-analysis was performed to derive a more precise estimation of the involvement of *HOTAIR* polymorphisms in cancer development. Data from cases (*n* = 7,772) and controls (*n* = 9,075) were extracted from eligible studies (*n* = 10) identified in a comprehensive literature search conducted in PubMed, Embase, and the Web of Science databases through January 20, 2016. Overall, association between polymorphism *rs920778* and increased cancer risk was significant in allele contrast (odds ratio (OR) = 1.239, 95% confidence interval (CI) = 1.032 - 1.487) and recessive models (OR = 1.614, 95% CI = 1.082 - 2.406). In subgroup analysis based on ethnicity, a significant association between polymorphism *rs920778* and cancer susceptibility was observed in Asians under all models, but was most compelling under recessive (OR = 2.128, 95% CI = 1.417 - 3.197) and homozygous models (OR = 2.764, 95% CI = 2.221 - 3.440). Subgroup analysis by cancer type revealed a significant association between polymorphism *rs4759314* and susceptibility to gastric cancer in allele contrast (OR = 1.262, 95% CI = 1.073 - 1.486), dominant (OR = 1.280, 95% CI = 1.060 - 1.547), and heterozygous models (OR = 1.288, 95% CI = 1.057 - 1.570). In conclusion, the results indicated that *HOTAIR* polymorphism *rs920778* was more generally associated with cancer risk, particularly in Asians, while *rs4759314* was a risk factor for gastric cancer.

## INTRODUCTION

Long non-coding RNAs (lncRNAs) are defined as transcribed RNA molecules that are longer than 200 nucleotides and not translated into proteins [1]. Although their function was initially unclear, lncRNAs are now known to have critical roles in the regulation of gene expression through transcription, processing of small RNAs, and epigenetic modification as well as other regulatory functions [2, 3]. As key components in gene regulatory complexes, lncRNAs contribute to the activation or inhibition of expression of genes involved in diverse normal cellular processes, such as proliferation and apoptosis. Many of these same processes are corrupted in cancer, and thus deregulated lncRNA expression has been linked to development of the disease [3–6].

Increasing evidence indicates that one of these molecules, *HOX* transcript antisense intergenic RNA (*HOTAIR*), has an oncogenic role in the development of human cancer in diverse tissues [5, 7, 8]. *HOTAIR* is a 2158-nucleotide lncRNA transcribed from the antisense strand of the *HOXC* gene which is located on chromosome 12 [9]. *HOTAIR* represses transcription, but surprisingly, in trans at the *HOXD* gene cluster on chromosome 2 [8, 10]. The lncRNA has been proposed to function as a molecular scaffold for the assembly of polycomb repressive complex 2 (PRC2) and lysine specific demethylase 1/REST corepressor 1/RE1-silencing transcription factor (LSD1/CoREST/REST) complex at 5′ and 3′ domains, respectively. Histone H3K27 methylation and H3K4 demethylation activities are thus effectively localized [11], which ultimately results in efficient chromosome condensation and transcriptional repression of targeted genes [7].

Numerous studies have demonstrated that overexpression of *HOTAIR* occurs in many cancers. It is furthermore associated with poor prognosis, and in experimental models, it promotes tumor progression, invasion, and metastasis [12–16]. Finally, *HOTAIR* single nucleotide polymorphisms (SNPs) have been investigated as potential cancer susceptibility loci and linked to increased risk for human cancers, such as breast [17–19], esophageal squamous cell carcinoma [20], gastric [21–24], lung [25], and colorectal cancers [26]. However, the results remain controversial possibly due to the fact that independent studies are underpowered and biased, especially for small cohorts. Here, a meta-analysis of eligible studies conducted before January 20, 2016 was performed in order to obtain more precise and comprehensive insight into the impact of *HOTAIR* polymorphisms on cancer susceptibility. The results indicated that *HOTAIR* polymorphisms are associated with increased cancer risk, but mainly in stratified analysis based on ethnicity and cancer type.

## RESULTS

### Study characteristics

Our database search yielded 10 studies with a total of 7,772 cases and 9,075 controls that were eligible for our meta-analysis [17–26]. The main features of the eligible studies, which included genotyping method, are listed in Table 1. All were case-control studies and were comprised of individuals of Asian (*n* = 7) and Turkish descent (*n* = 3). In addition, the studies covered diverse tumor types: gastric cancer (*n* = 4) [21–24], breast cancer (*n* = 3) [17–19], colorectal cancer (*n* = 1) [26], lung cancer (*n* = 1) [25], and esophageal squamous cell carcinoma (*n* = 1) [20]. Quality of the included studies was assessed using the Newcastle Ottawa Scale, and all the studies scored a 7 or above (high-quality).

**Table 1:** Characteristics of studies on association between *HOTAIR* polymorphisms and cancers

Author	Year	Ethnicity	Cases	Controls	Source of Controls	Cancers	Single Nucleotide Polymorphisms	Genotyping Method	Quality Score
Gong	2016	Chinese	498	213	HB	Lung cancer	*rs4759314, rs7958904, rs1899663*	MALDI-TOF mass spectrometry	7
Yan	2015	Chinese	502	504	PB	Breast cancer	*rs1899663, rs4759314, rs920778*	PCR-RFLP, CRS-RFLP	8
Xue	2015	Chinese	1734	1855	HB	Colorectal cancer	*rs4759314, rs7958904, rs874945*	TaqMan	7
Pan	2015	Chinese	800	1600	HB	Gastric cancer	*rs920778, rs1899663, rs4759314*	PCR-RFLP	7
Guo	2015	Chinese	515	654	HB	Gastric cardia adenocarcinoma	*rs12826786, rs4759314, rs10783618*	PCR-RFLP	7
Du	2015	Chinese	1275	1646	HB	Gastric cancer	*rs4759314, rs7958904, rs874945*	TaqMan	8
Bayram	2015	Turkish	123	122	HB	Breast cancer	*rs12826786*	TaqMan	7
Bayram	2015	Turkish	104	209	HB	Gastric cancer	*rs920778*	TaqMan	7
Bayram	2015	Turkish	123	122	HB	Breast cancer	*rs920778*	TaqMan	7
Zhang	2014	Chinese	2098	2150	HB	Esophageal squamous cell carcinoma	*rs920778, rs1899663, rs4759314*	PCR-RFLP	8

PB, population-based; HB, hospital-based; PCR-RFLP, polymerase chain reaction-restriction fragment length polymorphism; CRS-RFLP, created-restriction-site PCR-RFLP.

The number of *HOTAIR* SNPs extracted from all eligible studies was 7. Of these, only 2 SNPs, *rs4759314* and *rs920778*, were reported in 5 or more studies and were thus the focus of the meta-analysis. The genotype distributions of *HOTAIR rs4759314* and *rs920778* SNPs are shown in Table 2.

**Table 2:** Genotype distributions of *HOTAIR* polymorphisms *rs4759314* and *rs920778*

*rs4759314*	AA genotype		AG genotype		GG genotype		*P* for HWE in controls
Study	Cases	Controls	Cases	Controls	Cases	Controls	
Yan (2015)	451	448	50	54	1	2	0.78
Xue (2015)	1528	1608	200	236	5	11	0.47
Pan (2015)	451	914	48	83	1	3	0.45
Guo (2015)	461	589	53	64	1	1	0.59
Du (2015)	1083	1464	186	172	6	8	0.23
Zhang (2014)	917	910	81	89	2	1	0.44
***rs920778***	**CC genotype**		**CT genotype**		**TT genotype**		***P* for HWE in controls**
**Study**	**Cases**	**Controls**	**Cases**	**Controls**	**Cases**	**Controls**	
Yan (2015)	12	18	151	190	339	296	0.06
Pan (2015)	420	980	321	575	59	45	< 0.01
Bayram (2015-May)	20	38	52	105	32	66	0.74
Bayram (2015-Jan)	31	15	52	66	40	41	0.14
Zhang (2014)	1091	1323	826	749	181	78	0.03

HWE, Hardy-Weinberg equilibrium.

### *HOTAIR* polymorphism *rs920778* is associated with a general susceptibility cancer

The first step in the analysis was therefore to determine whether either *rs4759314* or *rs920778* was associated with a general risk for cancer regardless of tissue origin. Analysis including all individuals from all eligible studies revealed that association between the polymorphism *rs920778* and increased cancer risk was statistically significant in allele contrast (T *vs* C, OR = 1.239, 95% CI = 1.032 - 1.487, *P* = 0.021) and recessive genotype models (TT *vs* CT+CC, OR = 1.614, 95% CI = 1.082 - 2.406, *P* = 0.019). However, no significant association between the polymorphism *rs4759314* and increased susceptibility to cancer in any genotype model was observed. The analysis thus revealed an association between the T allele or the TT genotype for polymorphism *rs920778* and increased risk for cancer.

### *HOTAIR* polymorphism *rs4759314* is associated with an increased risk for gastric cancer

**Figure 1:**
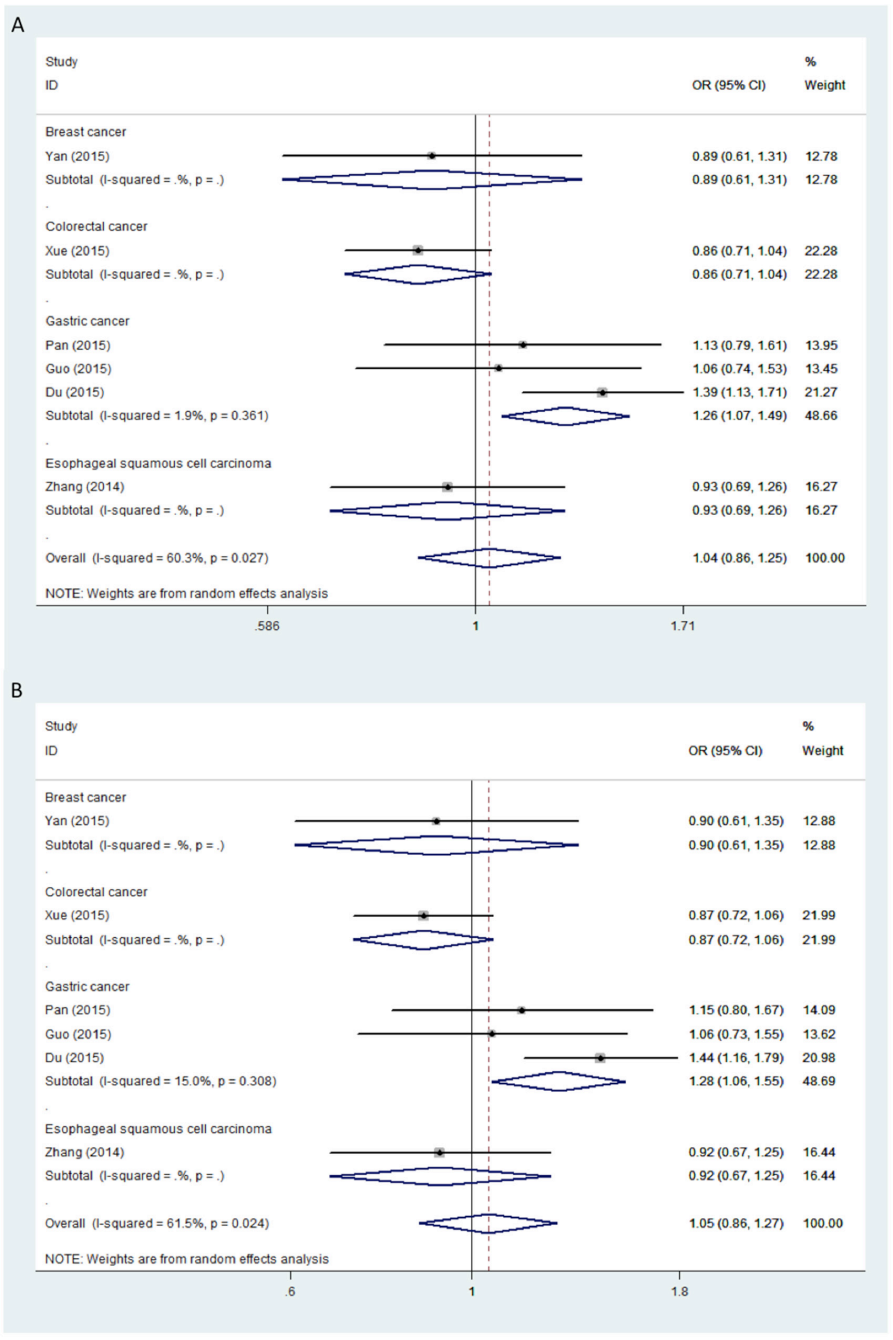
Forest plot of cancer risk in different cancer types associated with *HOTAIR* polymorphism *rs4759314* Models represented in **A.** allele contrast and **B.** dominant.

To determine whether *HOTAIR* polymorphisms *rs4759314* or *rs920778* were associated with risk for a specific cancer type, stratified analyses were performed on the basis of cancer tissue of origin. In this analysis, association of polymorphism *rs4759314* with increased gastric cancer susceptibility was statistically significant under allele contrast (G *vs* A, OR = 1.262, 95% CI = 1.073 - 1.486, *P* = 0.005), dominant (GG+AG *vs* AA, OR = 1.280, 95% CI = 1.060 - 1.547, *P* = 0.010), and heterozygous (AG *vs* AA, OR = 1.288, 95% CI = 1.057 - 1.570, *P* = 0.012) models (Figure 1). These results indicated that the G allele of *rs4759314* was linked to increased susceptibility specifically for gastric cancer.

Significant between-study heterogeneity was observed in allele contrast (I-square = 60.3%, *P* = 0.027), dominant (I-square = 61.5%, *P* = 0.024), and heterozygous models (I-square = 60.4%, *P* = 0.027). Therefore, the random-effects model was used to pool the data. In the stratified analysis based on cancer tissue of origin, no significant heterogeneity was found in the gastric cancer group.

### *HOTAIR* polymorphism *rs920778* is associated with cancer susceptibility in Asians

**Figure 2:**
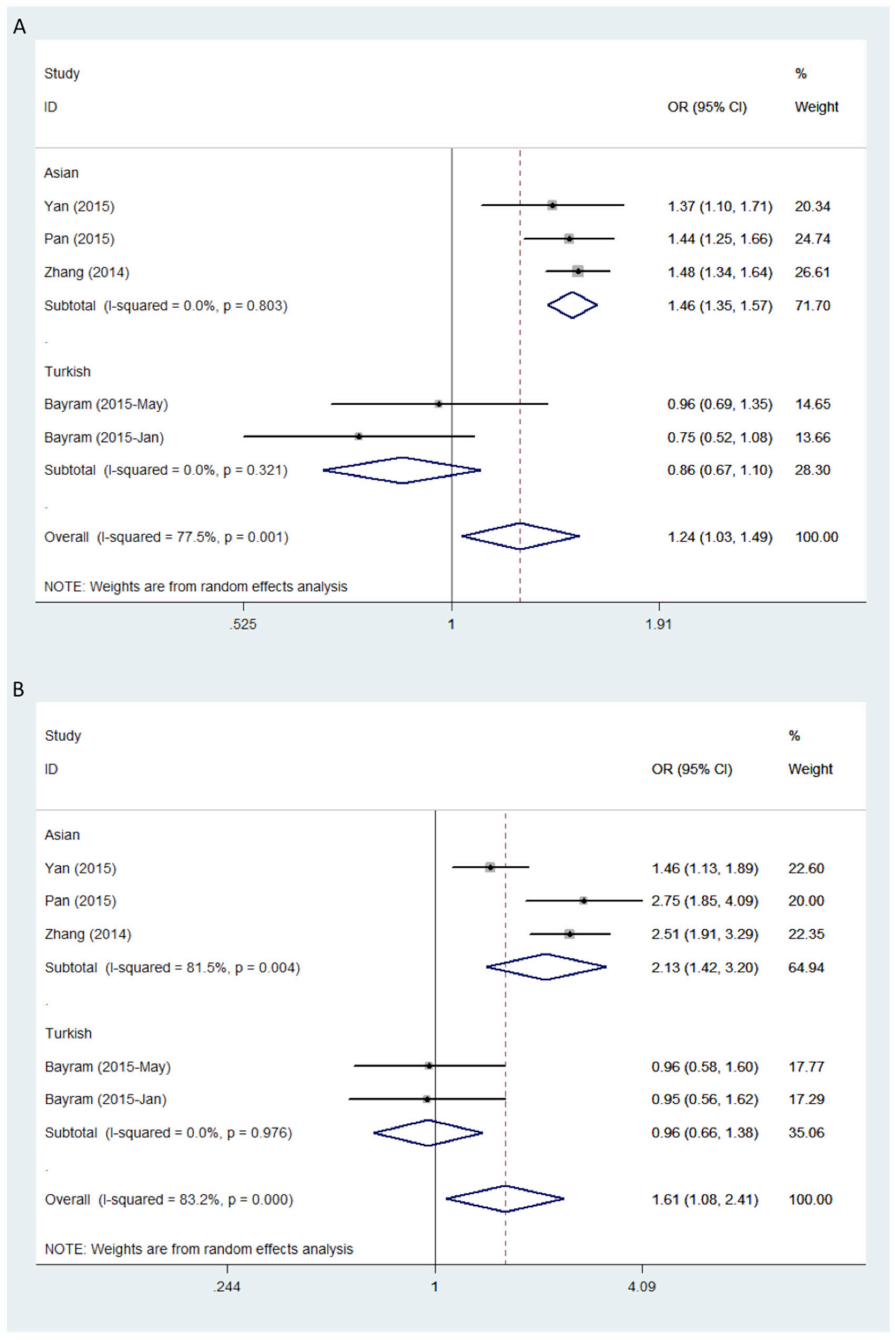
Forest plot of cancer risk in different ethnicities associated with *HOTAIR* polymorphism *rs920778* Models represented **A.** allele contrast and **B.** recessive.

The studies consisted of individuals of Asian and Turkish descent. Therefore associations between the polymorphisms and a general risk for cancer were examined on the basis of ethnicity. All individuals from studies involving *rs4759314* were Asian so analysis stratified by ethnicity was performed only for *rs920778*. A significant association between *rs920778* and cancer susceptibility was observed in Asians under all genetic models with the results as follows: allele contrast (T *vs* C, OR = 1.456, 95% CI = 1.349 - 1.571, *P* < 0.001); dominant model (TT+CT *vs* CC, OR = 1.462, 95% CI = 1.325 - 1.613, *P* < 0.001); recessive model (TT *vs* CT+CC, OR = 2.128, 95% CI = 1.417 - 3.197, *P* < 0.001); homozygous model (TT *vs* CC, OR = 2.764, 95% CI = 2.221 - 3.440, *P* < 0.001); and heterozygous model (CT *vs* CC, OR = 1.323, 95% CI = 1.194 - 1.466, *P* < 0.001) (Figure 2). The T allele or the TT genotype *rs920778* thus emerged as a potential genetic marker for increased cancer susceptibility especially in Asians.

Significant between-study heterogeneity was observed in all genetic models; therefore, analysis using the random-effects model was conducted on data stratified by ethnicity. Under these conditions, statistically significant heterogeneity was no longer observed within Asian or Turkish groups.

### Sensitivity analysis

The stability of the results of the meta-analysis was assessed in sensitivity analysis, where the effect of each study on the pooled OR was examined by repeating the meta-analysis after sequentially omitting each study. Through the deletion of only a single study which was based on a Turkish population [18], an association of the polymorphism *rs920778* with cancer risk emerged under dominant, homozygous, and heterozygous models. The *P*-values were not highly significant, however (data not shown). Therefore, the results of this meta-analysis including all eligible studies were generally and statistically stable.

### Publication bias

**Figure 3:**
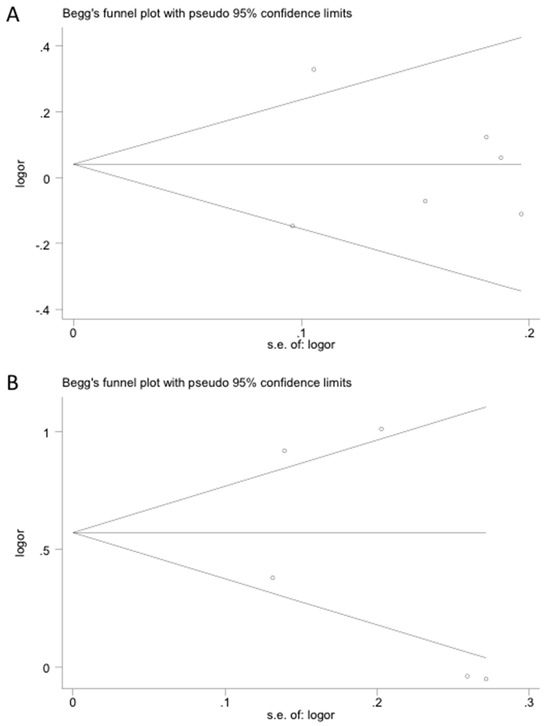
Publication bias tested by Begg's funnel plot Polymorphisms represented in **A.** *rs4759314* and **B.** *rs920778*.

Publication bias for each polymorphism in each model was evaluated using both the Begg's and Egger's tests. No obvious asymmetry was observed in any of the Begg's funnel plots indicating that publication bias was generally not a factor influencing the results (Figure 3). All *P*-values from the Egger's test and the Begg's test are listed in Table 3. The values are consistent with the absence of significant publication bias in the analysis of cancer risk and polymorphisms in most genotype models except in the case of polymorphism *rs920778*. For this polymorphism, publication bias was apparent in the allele contrast model. However, analysis with the trim and fill method demonstrated that the results of our study did not significantly change even after adjusting for the publication bias.

**Table 3:** ORs and 95% CI for cancers and the *HOTAIR* polymorphisms under different genetic models

Genetic models	n	OR [95% CI]	*P* (OR)	Model (method)	I-square (%)	*P* (H)	*P* (Begg)	*P* (Egger)
*rs4759314*								
Allele contrast (G *vs* A)								
All	6	1.037 [0.863 - 1.246]	0.700	R (D-L)	60.3	0.027	1.000	0.903
Gastric cancer	3	1.262 [1.073 - 1.486]	0.005	R (D-L)	1.9	0.361	-	-
Dominant model (GG+AG *vs* AA)								
All	6	1.049 [0.864 - 1.275]	0.629	R (D-L)	61.5	0.024	1.000	0.868
Gastric cancer	3	1.280 [1.060 - 1.547]	0.010	R (D-L)	15.0	0.308	-	-
Recessive model (GG *vs* AG+AA)								
All	6	0.739 [0.395 - 1.381]	0.343	F (M-H)	0.0	0.880	0.707	0.495
Gastric cancer	3	0.933 [0.378 - 2.300]	0.880	F (M-H)	0.0	0.934	-	-
Homozygous model (GG *vs* AA)								
All	6	0.747 [0.400 - 1.396]	0.361	F (M-H)	0.0	0.863	0.707	0.532
Gastric cancer	3	0.968 [0.393 - 2.387]	0.944	F (M-H)	0.0	0.931	-	-
Heterozygous model (AG *vs* AA)								
All	6	1.059 [0.872 - 1.286]	0.566	R (D-L)	60.4	0.027	1.000	0.833
Gastric cancer	3	1.288 [1.057 - 1.570]	0.012	R (D-L)	19.1	0.291	-	-
*rs920778*								
Allele contrast (T *vs* C)								
All	5	1.239 [1.032 - 1.487]	0.021	R (D-L)	77.5	0.001	0.027	0.030
Asian	3	1.456 [1.349 - 1.571]	< 0.001	R (D-L)	0.0	0.803	-	-
Dominant model (TT+CT *vs* CC)								
All	5	1.199 [0.916 - 1.570]	0.186	R (D-L)	73.2	0.005	0.221	0.170
Asian	3	1.462 [1.325 - 1.613]	< 0.001	R (D-L)	0.0	0.953	-	-
Recessive model (TT *vs* CT+CC)								
All	5	1.614 [1.082 - 2.406]	0.019	R (D-L)	83.2	<0.001	0.462	0.442
Asian	3	2.128 [1.417 - 3.197]	< 0.001	R (D-L)	81.5	0.004	-	-
Homozygous model (TT *vs* CC)								
All	5	1.549 [0.843 - 2.846]	0.159	R (D-L)	85.8	<0.001	0.086	0.078
Asian	3	2.764 [2.221 - 3.440]	< 0.001	R (D-L)	0.0	0.403	-	-
Heterozygous model (CT vs CC)								
All	5	1.115 [0.862 - 1.441]	0.407	R (D-L)	67.8	0.014	0.221	0.143
Asian	3	1.323 [1.194 - 1.466]	< 0.001	R (D-L)	0.0	0.938	-	-

OR, Odds ratio; CI, confidence intervals; *P* (H), *P* for heterogeneity; n, number of included studies; F, fixed-effect model; R, random-effects model; M-H, Mantel-Haenszel method; D-L, DerSimonian-Laird method.

## DISCUSSION

The overexpression of *HOTAIR* in various cancer types has led to its examination as a candidate molecule for the diagnosis and treatment of the disease. Here, *HOTAIR* was investigated for potential links to cancer susceptibility. We performed a meta-analysis using data from available cases (*n* = 7,772) and controls (*n* = 9,075) in the literature to date to provide further evidence that *HOTAIR* polymorphisms *rs4759314* and *rs920778* are associated with cancer risk. Only polymorphism *rs920778* was found to be generally associated with increased risk for cancer, but statistically significant associations for both polymorphisms were revealed when data were stratified based on ethnicity and cancer type.

The results of our analysis indicated that the T allele or TT genotype of polymorphism is a potential genetic marker for cancer susceptibility, especially in Asians, and that the G allele in polymorphism *rs4759314* might be related to susceptibility for gastric cancer. A possible mechanism underlying the association of these alleles to increased susceptibility is through the regulation of the expression of *HOTAIR* itself. Polymorphism *rs4759314* resides in an intronic promoter region, which was found to influence the activity of this promoter and expression of *HOXC11* gene [23]. For polymorphism *rs4759314*, increased *HOTAIR* expression originated from the G (relative to the A) allele in luciferase reporter assays. A similar finding was made for polymorphism *rs920778*, which resides in a novel *HOTAIR* intronic enhancer. In this case, increased *HOTAIR* expression was associated with T allele carriers [20, 22]. For both polymorphisms, the results of the functional assay correlated with the results of our meta-analysis; higher risk alleles drove increased expression of *HOTAIR*.

A sufficient number of cases and controls were pooled from different studies and provided a more accurate estimation of the associations between the *HOTAIR* polymorphisms and cancer risk as compared to individual studies. However, some limitations of this meta-analysis exist. First, although the analysis was performed with strict criteria for study inclusion and precise data extraction, significant between-study heterogeneity existed in some comparisons. However, after analysis stratified by cancer type or ethnicity, heterogeneity between the subgroups was significantly reduced. Second, our analysis was limited to individuals of Asian and Turkish descent so that it remains unclear as to whether these results can be generalized to other populations.

In conclusion, our results indicate that *HOTAIR* polymorphism *rs920778* is more generally associated with cancer risk, particularly in Asians, whereas polymorphism *rs4759314* may be a risk factor for gastric cancer. However, these polymorphisms require further evaluation in future well-designed studies as potential genetic susceptibility loci in different cancers as well as ethnic populations.

## MATERIALS AND METHODS

### Search strategy

PubMed, Embase, and Web of Science databases were searched for studies reporting association of *HOTAIR* polymorphisms with cancer risk up to the date of January 20, 2016. The literature search was performed using free-text words combined with Medical Subject Headings (MeSH), such as “Neoplasms”, “*HOTAIR* long untranslated RNA, human” and “Polymorphism, Single Nucleotide”. Gene-specific terms (*HOX* transcript antisense intergenic RNA or *HOTAIR*) were combined with polymorphism-specific terms (polymorphism or polymorphisms or variation or variations or variant or variants or mutation or mutations or genotype or genotypes) and disease-specific terms (cancer or cancers or tumor or tumors or neoplasm or neoplasms) to retrieve eligible studies. References cited in retrieved articles were also reviewed to identify additional potentially relevant studies.

### Inclusion and exclusion criteria

Inclusion criteria for studies were the following: (1) case-control or cohort study design; (2) evaluating associations between *HOTAIR* polymorphisms and all types of cancer; (3) providing sufficient data for SNP allele and genotype frequencies; (4) published in English; and (5) performed on humans. Exclusion criteria were the following: (1) reviews and comments; (2) performed on animals, and (3) duplication of a previous publication.

### Data extraction

Two investigators (QQ and JW) independently extracted the following data from each study: first author's surname, publication year, ethnicity, cancer types, numbers of cases and controls, and genotype distributions of cases and controls. Different ethnicity descents were categorized as Asian or Turkish. Study design was stratified into population-based and hospital-based studies. The results were compared and disagreement was resolved by discussion with a third reviewer (BH) until consensus was reached.

### Quality assessment

The Newcastle-Ottawa Scale and Agency for Healthcare Research and Quality (http://www.ohri.ca/programs/clinical_epidemiology/oxford.asp; maximum score = 9 points) was used to evaluate the methodological quality, which scored studies based on the selection of patients, the comparability of the groups, and the quality of the sampling process. A study awarded a score of 0 - 3, 4 - 6, or 7 - 9 was considered as a low-, moderate-, or high-quality study, respectively.

### Statistical analysis

To obtain a more comprehensive assessment of associations between *HOTAIR* polymorphisms and cancer susceptibility, five different comparison models were used: allele contrast, dominant, recessive, homozygous and heterozygous. Odds ratio (OR) with 95% confidence intervals (CI) was used to estimate the strength of associations, and the significance of ORs was determined with the Z test. Heterogeneity among the included studies was assessed by Chi square-based Q statistic. A random-effects (DerSimonian-Laird method) or fixed-effect (Mantel-Haenszel method) model was used to calculate pooled effect estimates in the presence (*P* < 0.05) or absence (*P* > 0.05) of heterogeneity. The method of subgroup analysis according to the ethnicity or cancer type of the participants was also applied to gain more precise results. Sensitivity analysis was conducted by sequentially excluding each study. The Begg's test and the Egger's test were performed to analyze for the presence of publication bias. If any possible bias was observed, the trim and fill method was used to identify and adjust for those studies. Data analyses were carried out using Stata software, version 11.0 (Stata Corporation; College Station, TX, USA). *P*-values < 0.05 were considered statistically significant.
